# Data on partial polyhydroxyalkanoate synthase genes *(phaC)* mined from *Aaptos aaptos* marine sponge-associated bacteria metagenome

**DOI:** 10.1016/j.dib.2017.11.011

**Published:** 2017-11-08

**Authors:** Tan Suet May Amelia, Al-Ashraf Abdullah Amirul, Kesaven Bhubalan

**Affiliations:** a School of Marine and Environmental Sciences, Universiti Malaysia Terengganu, 21030 Kuala Nerus, Terengganu, Malaysia; b School of Biological Sciences, Universiti Sains Malaysia, 11800 Pulau Pinang, Malaysia; c Malaysian Institute of Pharmaceuticals and Nutraceuticals, NIBM, 11700 Pulau Pinang, Malaysia; d Centre for Chemical Biology, Sains@USM, First Floor, Block B, 10, Persiaran Bukit Jambul, 11900 Bayan Lepas, Penang, Malaysia; e Institute of Marine Biotechnology, Universiti Malaysia Terengganu, 21030 Kuala Nerus, Terengganu, Malaysia

**Keywords:** Marine sponge, Metagenome, Polyhydroxyalkanoate synthase gene, Phylogenetic analysis

## Abstract

We report data associated with the identification of three polyhydroxyalkanoate synthase genes *(phaC)* isolated from the marine bacteria metagenome of *Aaptos aaptos* marine sponge in the waters of Bidong Island, Terengganu, Malaysia. Our data describe the extraction of bacterial metagenome from sponge tissue, measurement of purity and concentration of extracted metagenome, polymerase chain reaction (PCR)-mediated amplification using degenerate primers targeting Class I and II *phaC* genes, sequencing at First BASE Laboratories Sdn Bhd, and phylogenetic analysis of identified and known *phaC* genes. The partial nucleotide sequences were aligned, refined, compared with the Basic Local Alignment Search Tool (BLAST) databases, and released online in GenBank. The data include the identified partial putative *phaC* and their GenBank accession numbers, which are *Rhodocista* sp. *phaC* (MF457754), *Pseudomonas* sp. *phaC* (MF437016), and an uncultured bacterium AR5-9d_16 *phaC* (MF457753).

**Specifications Table**

**Table:** 

Subject area	Biology
More specific subject area	Molecular Biology, Bioinformatics
Type of data	Table, figure.
How data was acquired	Spectrophotometry (Nanodrop™ 2000, Thermo Fisher),
	PCR (Applied Biosystems™ Veriti 96-Well Thermal Cycler, Thermo Fisher), Electrophoresis (PowerPac™ Basic power supply, Bio-Rad),
	DNA Sequencing (First BASE Laboratories Sdn Bhd),
	Basic Local Alignment Search Tool (BLAST),
	Molecular Evolutionary Genetics Analysis Version 7.0.20 (MEGA7),
	and GenBank.
Data format	Filtered and analysed
Experimental factors	The putative Class I and II *phaC* genes were isolated with semi-nested PCR using specific *phaC* primers:
	CF1 forward primer:
	5′-ATCAACAA(A/G)T(A/T)CTAC(A/G)TC(C/T)T(C/G)GACCT-3′
	CF2 forward primer:
	5′-GT(C/G)TTC(A/G)T(C/G)(A/G)T(C/G)(A/T)(C/G)CTGGCGCAACCC-3′
	CF4 reverse primer:
	5′-AGGTAGTTGT(C/T)GAC(C/G)(A/C)(A/C)(A/G)TAG(G/T)TCCA-3′
Experimental features	Analysis was done with BLAST, BioEdit 7.2.6, Clustal W, MEGA 7.0.20.
Data source location	Bidong Island, Terengganu, Malaysia
Data accessibility	Partial nucleotide sequences are in the public repository of GenBank.
	GenBank accession number and URL:
	MF457753 (https://www.ncbi.nlm.nih.gov/nuccore/MF457753),
	MF457754 (https://www.ncbi.nlm.nih.gov/nuccore/MF457754),
	MF437016 (https://www.ncbi.nlm.nih.gov/nuccore/MF437016).
	All other data are within this article.

**Value of the data**

• This data reveals the presence of non-cultivable PHA-producing bacteria in marine sponges.
• This data can be used for comparative studies related to *phaC* isolated from marine environment especially marine sponges, which is known as a bacteria hot-spot.
• This data can be used for further experiments to provide insight into the enzymatic activity of the identified *phaC* to synthesise polyhydroxyalkanoate using model organisms, such as *E. coli*.
• This data serve as a benchmark as the first report of the isolation of *phaC* genes from the South China Sea sponge *Aaptos aaptos*.

## 1 Data

**Fig. 1:**
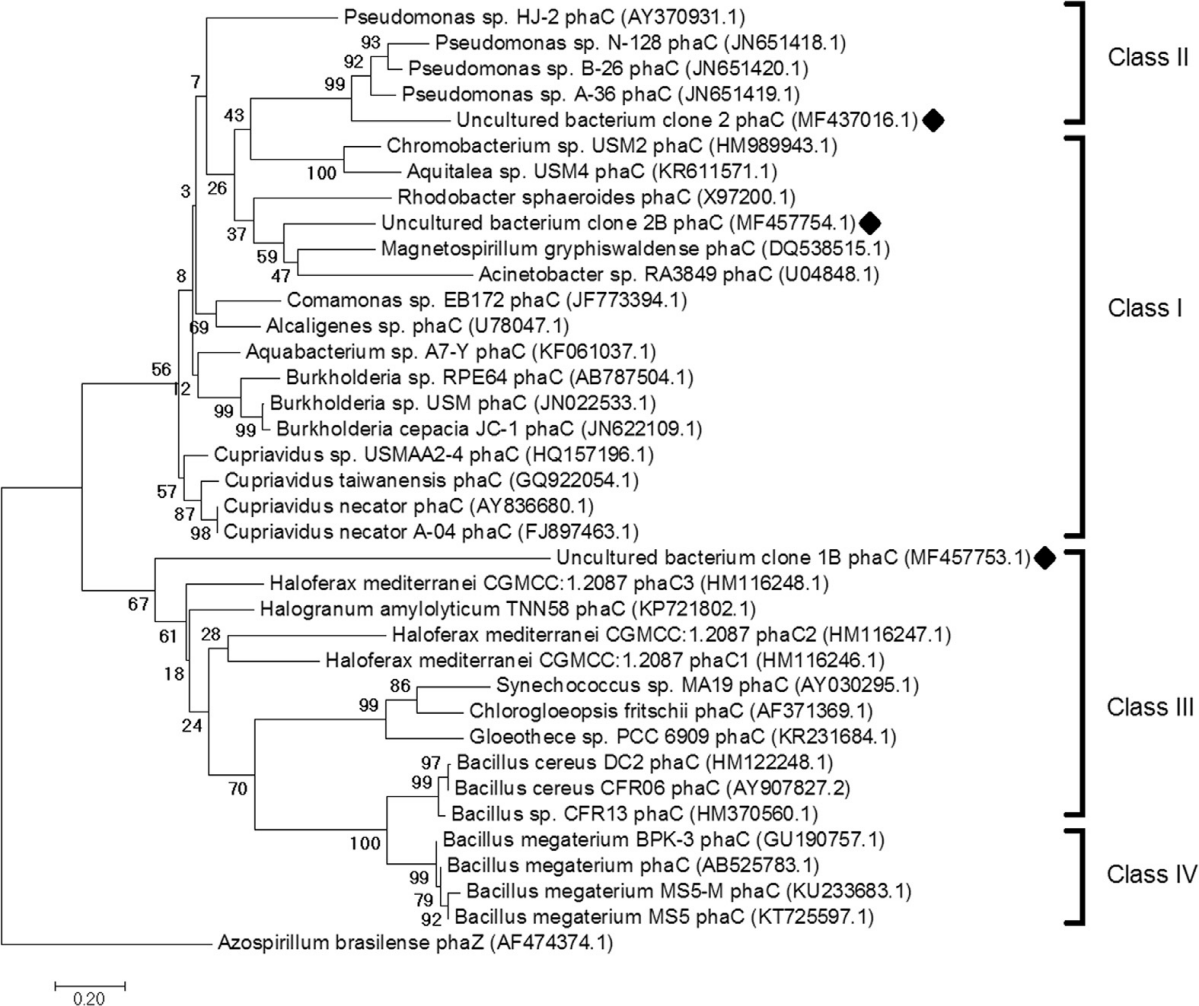
Diagram of Neighbour-Joining phylogenetic analysis constructed using MEGA7 software on the identified *phaC* genes with the complete coding sequences (CDS) of known *phaC* genes computed using Maximum Composite Likelihood method and 3000 bootstrap replicates, rooted on the outgroup from. Symbol (♦) represents the *phaC* genes identified in this data article.

These data provide detailed information on the isolation and identification of *phaC* from marine bacteria metagenome in *Aaptos aaptos* sea sponge at Bidong Island, Terengganu, Malaysia. Table 1 shows tabular data on the similarity comparison of sequenced *phaC* genes against the BLAST sequence databases. Table 2 shows data on the nucleotide sequences of the three putative, partial *phaC* genes identified from *A. aaptos* marine sponge-associated bacteria metagenome. The protein identifiers assigned by GenBank to uncultured bacterium *phaC* 2 and 2B are ASV71961.1 and ASY93340.1 respectively. Fig. 1 shows a phylogenetic Neighbour-Joining tree on the evolutionary relationships of identified and known *phaC* genes from variable sources.

**Table 1:** Accession number and similarity of identified *phaC* genes against the BLAST sequence databases.

Isolated Gene	Closest BLAST hit	Similarity
Uncultured bacterium clone 2 *phaC* (MF437016)	Pseudomonas stutzeri 1317 *phaC* (AY278219.1)	88%
Uncultured bacterium clone 1B *phaC* (MF457753)	Uncultured bacterium AR5-9d_16 *phaC* (AB220790.1)	93%
Uncultured bacterium clone 2B *phaC* (MF457754)	Rhodocista pekingensis *phaC* (AY283802.1)	74%

**Table 2:** Nucleotide sequences of the three putative, partial *phaC* genes identified from *A. aaptos* marine sponge-associated bacteria metagenome.

Isolated Gene	Accession Number	Nucleotide Sequence
Uncultured bacterium clone 2 *phaC*	MF437016	5′-CGTTCAATCAGCGGCAGCAAGGAGGTCAACCTGCTCGGCGCCTGTGCCGGCGGTCTGACCATCGCGGCCCTGCAGGGCCACCTGCAAGCCAAACGGCAATTGCGCAAGGTTGGCTGCGCCACCTATCTAGTCAGCCTGATGGACGCCCAGGTAGAAAGCCCTGCGATGCTGTTCGCAGATGAGCAGACCCTGGAGTCGGCCAAGCGCCGCTCCTACCAGAGCGGAGTGCTGGACGGCGGCGACATGGCTCGGGTGTTCGCCTGGATGCGCCCCAACGACCTGATCTGGAACTATGTGGTCAACAACTACCTA-3′
Uncultured bacterium clone 1B *phaC*	MF457753	5′-ACTAGTTGTCTACTACTGCCTATCAACAAGTTCTACGTCTTGGACCTATCAACAAGTTCTACGTCTAGGACCTATTGGGTCGTCCTCTACCTAGGAACAAGTTCTACGTCTTGGACCTATGAACAAGTTCTACGTCTAGGACCTATTGGGTCGTCCTCTACCTAGGACCTAGTTCTACGTCTTGGACCTATGAACAAGTTCTACGTCTTGGACCTATTGCCACGTCCTCTACCTAGGACCTAGTTCTACGTCTTGGACCTATGACCAAGTTCTACGTCTAGGACCTAGGACCACGTCCTCCACCTAGGACCTATTTGGACGTCCTGGACCTAGGAACAAGTTCTACATCTTGGACCTATGGGCAAGTTATCCACCTAGGACCTATTGCCACGTCTTGGTGCTAGGACCAAGTTCTACGTCCTGGACCTAGGGGGGAGTCCAACATCTAGAAATA-3′
Uncultured bacterium clone 2B *phaC*	MF457754	5′-TTGTGTTCATCATGTCCTGGCGCAACCCGGACGCCAAGCTCGCGGACAATGCGTTCGAAGACTACATGGCGGAGGGGCCGCTGGCGGCGCTGGAAGCCATCGAGGCAGCGACAGCCGAGCGGGAGGTCAACGCCGTGGGTTATTGCATCGGCGGCACGCTGATGGCGGCGACGCTGGCCTGGATGGCGGCCAGGGACGACGAGCGCGTCAAGAGCTCCACTTTCCTGTCCACCAGGGTGGATTTCGAGGAGGCAGGCGACCTCGGCGTGTTCCTGGACGAGGCGCAGCTGGGAGCGCCGGAGGAGCGGACGCGCGAGGACGGCTTCTGGCGGCGCGGAGATGGCGGCGACCTTCAGCGGGATGCGCGCGAACAACCTGAT-3′

## 2 Experimental design, materials and methods

The marine bacteria metagenome was extracted from the tissue of the sea sponge *Aaptos aaptos*, which was collected in the waters of Bidong Island, Terengganu, Malaysia at a depth of 15 m (GPS: 5°36'48.1" N 103°03'30.0" E) on June 16, 2016. The metagenome was extracted from 1 cm^3^ sponge tissue using phenol-chloroform isoamyl alcohol (PCI) according to modified protocols by Beloqui and co-workers [1]. Whole genome amplification (WGA) was then carried out on the extracted metagenome using REPLI-g Mini Kit (Qiagen). The purity and concentration of the metagenome before and after WGA were measured using Nanodrop™ 2000 Spectrophotometer (Thermo Fisher Scientific). The reaction mixture for PCR was prepared using EconoTaq® PLUS 2X Master Mix (Lucigen) according to the manufacturer's instructions prior to the PCR amplification process, which was proceeded in the sequence of pre-denaturation at 95 °C for 3 min, denaturation at 95 °C for 30 s, annealing at 56 °C for 1 min, extension at 72 °C for 90 s, and final extension at 72 °C for 5 min using Applied Biosystems™ Veriti 96-Well Thermal Cycler (Thermo Fisher Scientific). The degenerate primers that targeted the Class I and II *phaC* genes were applied in the PCR process, which were forward primer, CF1 (5′-ATCAACAA(A/G)T(A/T)CTAC(A/G)TC(C/T)T(C/G)GACCT-3′), and reverse primer, CR4 (5′-AGGTAGTTGT(C/T)GAC(C/G)(A/C)(A/C)(A/G)TAG(G/T)TCCA-3′) [2]. A semi-nested PCR was then carried out using forward primer, CF2 (5′-GT(C/G)TTC(A/G)T(C/G)(A/G)T(C/G)(A/T)(C/G)CTGGCGCAACCC-3′), and reverse primer, CR4, with similar protocols to amplify the target gene. The amplified PCR product was separated by 0.7% w/v agarose gel electrophoresis [3] using PowerPac™ Basic power supply (Bio-Rad Laboratories), and visualised using Gel Doc™ EZ Imager (Bio-Rad Laboratories). The amplified *phaC* genes were sequenced via submission to First BASE Laboratories Sdn Bhd, which used Applied Biosystems™ Genetic Analyzer with Sanger sequencing method, prior to alignment and refinement using BioEdit software 7.2.6. The query sequences were compared against the sequence databases using the BLAST tool (Table 1). The sequences were then released in the GenBank nucleotide sequence databases on September 4, 2017, under accession numbers MF457754, MF457753, and MF437016 (Table 2).

The phylogenetic tree (Fig. 1) shows the evolutionary relationship among the three identified, putative partial *phaC* genes and previously reported *phaC* genes with complete cds (coding sequence) released in GenBank database, comprising of 36 ingroup nucleotide sequences and 1 outgroup nucleotide sequence, which was constructed using the Neighbour-Joining method [4]. The optimal tree with the sum of branch length=10.39764912 is shown. The percentage of replicate trees in which the associated taxa clustered together in the bootstrap test (3000 replicates) are shown next to the branches [5]. The tree is drawn to scale, with branch lengths in the same units as those of the evolutionary distances used to infer the phylogenetic tree. The evolutionary distances were computed using the Maximum Composite Likelihood method [6] and are in the units of the number of base substitutions per site. All positions containing gaps and missing data were eliminated. There were total 19 positions in the final dataset. Evolutionary analyses were conducted in MEGA7 [7].
